# Supplementary material for: Comparative genomics provides new insights into the diversity, physiology, and sexuality of the only industrially exploited tremellomycete: Phaffia rhodozyma
Source: BMC Genomics. 2016 Nov 9;17:901. doi: 10.1186/s12864-016-3244-7 (PMC5103461; doi:10.1186/s12864-016-3244-7)
Supplement: Additional file 6: — List of orphan genes with links to PFAM (related to Additional file 1: Table S1). (ZIP 1428 kb) [file 12864_2016_3244_MOESM6_ESM.zip › PFAM_UNIQUE/G04057_P.html]

#                                                                            --- full sequence --- -------------- this domain -------------   hmm coord   ali coord   env coord
  
# target name        accession   tlen query name           accession   qlen   E-value  score  bias   #  of  c-Evalue  i-Evalue  score  bias  from    to  from    to  from    to  acc description of target
  
#------------------- ---------- ----- -------------------- ---------- ----- --------- ------ ----- --- --- --------- --------- ------ ----- ----- ----- ----- ----- ----- ----- ---- ---------------------
  
G04057\_P             -            316 AF1Q                 PF15017.1     88       2.6    6.7   5.4   1   3         4   3.4e+03   -3.3   0.0    62    70    87    95    70   104 0.58 - Drug resistance and apoptosis regulator  
G04057\_P             -            316 AF1Q                 PF15017.1     88       2.6    6.7   5.4   2   3    0.0013       1.1    7.9   2.1    25    66   141   182   135   206 0.80 - Drug resistance and apoptosis regulator  
G04057\_P             -            316 AF1Q                 PF15017.1     88       2.6    6.7   5.4   3   3       2.1   1.8e+03   -2.4   0.0    62    72   287   297   269   301 0.57 - Drug resistance and apoptosis regulator  
G04057\_P             -            316 Caldesmon            PF02029.10   492       3.6    4.5   9.0   1   1      0.04       4.2    4.3   9.0    76   165   133   220    79   302 0.73 - Caldesmon  
G04057\_P             -            316 DUF737               PF05300.6    187       6.5    5.3   7.7   1   2      0.65   3.2e+02   -0.2   0.2    44    75    70   101    18   114 0.60 - Protein of unknown function (DUF737)  
G04057\_P             -            316 DUF737               PF05300.6    187       6.5    5.3   7.7   2   2     0.002         1    8.0   2.6    24   104   146   226   124   244 0.91 - Protein of unknown function (DUF737)  
G04057\_P             -            316 Nop14                PF04147.7    840       8.7    2.8  12.0   1   1      0.18        13    2.2  12.0   298   391    87   200    78   251 0.61 - Nop14-like family  
G04057\_P             -            316 ORC6                 PF05460.8    353         4    5.1  13.1   1   1     0.033       8.9    3.9  13.1    90   166    16   147     3   299 0.71 - Origin recognition complex subunit 6 (ORC6)  
G04057\_P             -            316 SDA1                 PF05285.7    324      0.29    9.0  11.9   1   1    0.0036      0.37    8.7  11.9    92   216    92   242    76   298 0.58 - SDA1  
G04057\_P             -            316 Upf2                 PF04050.9    171       7.3    5.0  11.7   1   1     0.023        18    3.8  11.7    12   101   153   260   140   307 0.62 - Up-frameshift suppressor 2  
G04057\_P             -            316 Utp11                PF03998.8    243      0.75    8.2   5.1   1   1    0.0047       1.4    7.3   5.1   137   228   136   218   122   233 0.70 - Utp11 protein
